# The Outside-In Journey of Tissue Transglutaminase in Cancer

**DOI:** 10.3390/cells11111779

**Published:** 2022-05-29

**Authors:** Livia Elena Sima, Daniela Matei, Salvatore Condello

**Affiliations:** 1 Department of Molecular Cell Biology, Institute of Biochemistry of the Romanian Academy, 060031 Bucharest, Romania; lsima@biochim.ro; 2 Department of Obstetrics and Gynecology, Feinberg School of Medicine, Northwestern University, Chicago, IL 60611, USA; daniela.matei@northwestern.edu; 3 Robert H Lurie Comprehensive Cancer Center, Feinberg School of Medicine, Northwestern University, Chicago, IL 60611, USA; 4 Jesse Brown VA Medical Center, Chicago, IL 60612, USA; 5 Department of Obstetrics and Gynecology, Indiana University School of Medicine, Indianapolis, IN 46202, USA; 6 Simon Comprehensive Cancer Center, Indiana University School of Medicine, Indianapolis, IN 46202, USA

**Keywords:** tissue transglutaminase, cancer, fibronectin, integrin, tumor microenvironment, cancer stem cells, immune cells, therapy, extracellular matrix

## Abstract

Tissue transglutaminase (TG2) is a member of the transglutaminase family that catalyzes Ca^2+^-dependent protein crosslinks and hydrolyzes guanosine 5′-triphosphate (GTP). The conformation and functions of TG2 are regulated by Ca^2+^ and GTP levels; the TG2 enzymatically active open conformation is modulated by high Ca^2+^ concentrations, while high intracellular GTP promotes the closed conformation, with inhibition of the TG-ase activity. TG2’s unique characteristics and its ubiquitous distribution in the intracellular compartment, coupled with its secretion in the extracellular matrix, contribute to modulate the functions of the protein. Its aberrant expression has been observed in several cancer types where it was linked to metastatic progression, resistance to chemotherapy, stemness, and worse clinical outcomes. The N-terminal domain of TG2 binds to the 42 kDa gelatin-binding domain of fibronectin with high affinity, facilitating the formation of a complex with β-integrins, essential for cellular adhesion to the matrix. This mechanism allows TG2 to interact with key matrix proteins and to regulate epithelial to mesenchymal transition and stemness. Here, we highlight the current knowledge on TG2 involvement in cancer, focusing on its roles translating extracellular cues into activation of oncogenic programs. Improved understanding of these mechanisms could lead to new therapeutic strategies targeting this multi-functional protein.

## 1. Structure and Functions

**Figure 1:**
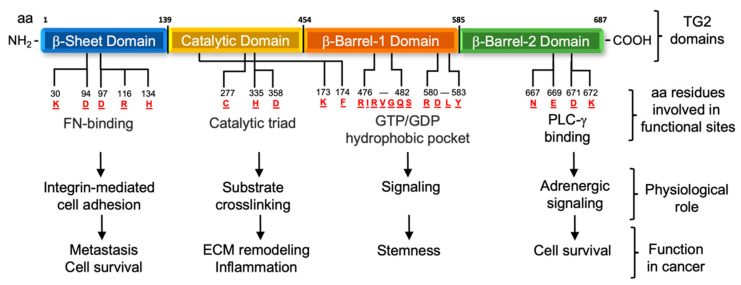
Functional domains of TG2. TG2 has four distinct domains. An N-terminal β-sandwich domain (amino acids (aa) residues: 1–139); a catalytic core domain (aa: 140–454); a β-barrel 1 domain that contains a GTP/ATP-binding site (aa: 456–585); and the C-terminal β-barrel 2 domain that can recruit and activate phospholipase C (aa: 586–687). The residues D94, D97 and more recently K30, R116, and H134 on the N-terminal domain are critical for the interaction with the 42 kDa gelatin-binding domain of FN. The catalytic triad C277, H335, D358 is responsible for the transamidating activity. The nucleotide-binding hydrophobic pocket is formed by the residues K173 and F174 located on the catalytic core and the residues R476, I477, R478, V479, G480, Q481, S482, R580, D581, L582, Y583 located on the β-barrel 1 and β-barrel 2 domains. The aa N667, E669, D671, K672 on the β-barrel 2 domain are responsible for the interaction with PLC-γ that supports signaling from adrenergic receptors. In cancer, the FN-binding domain is responsible for the integrin mediated cell adhesion which has been correlated with metastatic progression and cell survival. The catalytic domain has been correlated with ECM remodeling and inflammation, while the β-barrel 1 and 2 domains mediate intracellular signaling linked with cell survival and stemness.

Tissue transglutaminase (TG2) is a 76-kD protein belonging to the transglutaminase family, which includes TG1–7, Factor XIII and erythrocyte protein 4.2. They have similar restricted substrate specificity and distinct mechanisms of transcriptional regulation, leading to a tissue specific pattern of expression. The four domains of TG2 include a N-terminus β-sandwich domain which modulates its binding to fibronectin (FN), a catalytic domain including the active site triad C^277^H^335^D^358^, involved in the acyl-transfer function, and two β-barrel domains [1,2] (Figure 1). A GTP/GDP-binding site is located between the residues from the first and last strands (amino acids 476–482 and 580–583) of β-barrel 1 and two core domain residues (Lys-173 and Phe-174) that protrude on a loop to meet β-barrel 1 [3], suggesting that TG2 functions as a GTP-ase. However, TG2 does not have a classical switch region, characteristic of G-proteins, and it remains unclear whether and how GTP/GDP binding affects signaling. TG2 also interacts with phospholipase C gamma (PLCγ) through a region mapped at its C-terminus, supporting signaling from adrenergic receptors [4]. The functions of the protein are modulated through large allosteric changes in the protein structure, which are tightly regulated in biological systems. For example, the crystal structure of guanine-nucleotide bound TG2 (PDB ID 1KV3) showed a compact conformation where the 2 β-barrel domains folded over the catalytic triad, obstructing the accessibility of Cys^277^, while the structure of the enzymatically active TG2 (PDB ID 2Q3Z) showed an open, near-linear conformation exposing the catalytic core. In this “open” state, TG2 cannot bind GTP/GDP, but is able to interact with substrates for transamidation. On the contrary, high extracellular Ca^2+^ concentrations cause the protein to adopt an open structure and be enzymatically active [4]. Thus, the physiological functions of TG2 are regulated by the cellular context and localization. 

At the plasma membrane, the N-terminal domain of TG2 binds with high affinity (Kd ~8–10 nM) to the I_6_II_1,2_I_7–9_ modules representing the gelatin-binding domain of FN (FN42). The TG2/FN complex provides a binding site for β1 and β3 integrins [5,6,7]. Initially, the FN-binding region was thought to be represented by amino acids 88–106 forming a β hairpin loop [8]. However, more recent analyses based on hydrogen/deuterium exchange and mass spectrometry point to residues K30, R116, and H134 as key points of interaction with FN [9]. Aside from integrins, the TG2/FN complex recruits other membrane receptors such as the platelet derived growth factor receptor beta (PDGFR-β) [10,11] and the Frizzled-7 receptor [12]. As mutations in the catalytic core do not alter FN/integrin/TG2 complex formation, this role is independent of the transamidase function [6]. At the plasma membrane, TG2 also interacts with and stabilizes tubulin, microtubule-binding proteins [13] and vimentin, contributing to stress fibers formation [14].

Within the cytosol, where Ca^2+^ concentrations are low, most of the protein is thought to assume a transamidation-inactive, closed conformation and it is believed that TG2 binds to GTP and to other protein partners, altering cellular signaling [4,15]. However, it is possible that under certain conditions, localized calcium pools might exist that are sufficient to activate a subset of TG proteins.

Although it lacks a leader sequence, TG2 is secreted in the extracellular space where it remodels the matrix. It has been suggested that the enzyme concentrates at cell adhesion points rich in β1 integrin and gets externalized and distributed along the basal membrane [16]. Its externalization into the extracellular matrix (ECM) depends on its active enzymatic core and intact FN-binding domain [17]. In the ECM, where Ca^2+^ concentrations are high and nucleotide concentrations are low, TG2 functions as a transamidase, facilitating Ca^2+^-dependent incorporation of amines into proteins and acyl-transfer between glutamine and lysine residues on protein chains, leading to protein crosslinking and facilitating matrix remodeling [18,19]. Multiple matrix proteins are known to be TG2 substrates, including FN [20], fibrin [21], osteopontin [18], laminin [22], collagen [23,24], and others. These functions suggest that TG2 plays important roles in cell adhesion, migration, and stromal assembly, key processes during cancer progression. 

## 2. TG2 in Cancer

**Figure 2:**
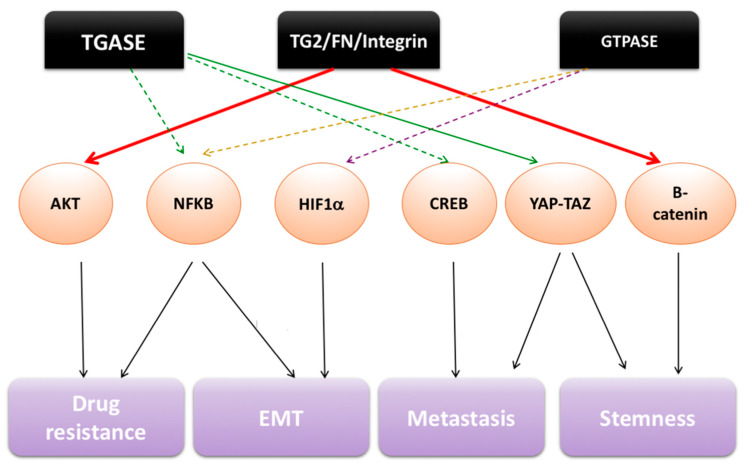
Mechanisms involving TG2 in cancer. TG2 is aberrantly expressed in several cancers where it modulates molecules involved in the activation of oncogenic pathways. TG-ase activity is linked to EMT, metastatic progression and drug resistance by regulating NF-κB, CREB, and YAP/TAZ signaling. By promoting integrin-mediated cell adhesion to FN, TG2 regulates β-catenin activation through a c-Src-dependent mechanism, leading to cancer cell proliferation and stemness. Active TG2/FN/β-Integrin complexes modulate PI3K/Akt pathway enhancing resistance to apoptosis induced by chemotherapy. As a GTP-ase, TG2 regulates NF-κB and HIF-1α signaling which are involved in EMT, drug resistance and stemness.

TG2 has been linked to cancer progression by many studies during the past fifteen years. The protein was found to be upregulated in glioblastoma [25], ovarian [26,27], pancreatic [28], lung [29] and breast cancer [30]. Furthermore, a correlation with poor clinical outcomes was found in pancreatic [31], ovarian [32] and lung cancer [33], supporting the concept that TG2 exerts a tumor promoter role. TG2 was shown to activate several oncogenic pathways, including the nuclear factor kappa-light-chain-enhancer of activated B cells/NF-κB, focal adhesion kinase/FAK, protein kinase B/Akt, β-catenin, Ras homolog family member A (RhoA), Yes-associated protein 1/YAP) which have been implicated in cancer progression [34,35,36,37,38] (Figure 2). In all, three themes have emerged. First, TG2 was shown to mediate chemotherapy and radiation-resistance [39] through activation of the NF-κB survival pathway [39,40] or through promotion of “outside-in” signaling initiated through TG2-regulated cellular adhesion to the matrix [31]. Second, TG2 has been linked to metastasis, with TG2 knockdown ovarian cancer (OC) cells causing less peritoneal dissemination in ovarian orthotopic xenograft models [26,27]. TG2 was found to induce epithelial–mesenchymal transition (EMT) [26,27] and to regulate cancer cell adhesion to the matrix [26]. These initial observations in OC models were validated in breast and lung cancer models [37,41]. Lastly, TG2 was identified as being highly expressed in cancer stem cells (CSCs) in ovarian [42], skin [43], breast [36], and brain cancer models [44] (Table 1). 

Although the consensus in the field is that TG2 promotes an oncogenic phenotype, the mechanism by which this occurs and which function of the protein is most important in the process remain disputed. We and others have shown that interactions with integrins and FN at the plasma membrane are essential to activating “outside-in” signaling (FAK, Akt, β-catenin, Src, epidermal growth factor receptor/EGFR) [34,45] and are clearly implicated in cancer metastasis and stemness. However, in other contexts, the transamidase function was linked to oncogenic activity: nuclear factor of kappa light polypeptide gene enhancer in B-cells inhibitor, alpha (IκBα) was shown to be crosslinked by TG2 leading to NF-κB activation [46]; RhoA was transamidated by TG2 in HeLa cells becoming constitutively active [47]. Lastly, the GTPase function was also implicated in the oncogenic phenotype. Studies using TG2 mutants (R580A which is unable to bind GTP and C277S which is enzymatically inactive) have implicated the nucleotide-binding function of TG2 in stemness [38,43]. Aside from its functions in cancer cells, TG2 is also expressed in stromal cells and influences tumor progression as we discuss in this review.

## 3. Intracellular Functions of TG2 in Tumor Cells

As outlined above, the enzyme is overexpressed in multiple solid tumors and involved in regulation of tumor progression, resistance to chemo- and radiotherapy and cancer stemness. Most of the protein is located in the cytosol of cancer cells, with a small proportion being present in the plasma membrane and in the nucleus. The intracellular roles of the protein revolve around the enzymatic and the GTPase functions, which are tightly modulated by Ca^++^ and GDP/GTP levels in tumor cells. Although the high-intracellular-GTP concentrations should inhibit the crosslinking function of TG2, there is evidence that under certain conditions, and particularly in neoplastic cells, the protein is enzymatically active. For example, some of the first reports recognizing the link between TG2 and cancer focused on activation of the survival pathway NF-κB. It was reported that the regulatory subunit IKBα is a direct substrate of TG2, gets crosslinked by the enzyme and is ubiquitinated for proteasomal degradation, allowing activation of Rel B and engagement of pro-inflammatory/survival genes [22]. Subsequently, activation of NF-κB plays an important role in inducing drug resistance and EMT.

The hallmark of EMT is the loss of E-cadherin, important to maintain cell–cell contacts, and the parallel increase in its transcriptional repressors Snail, Slug, Zeb 1/2, and Twist 1/2 regulated by tumor growth factor β1 (TGF-β1) [78]. Induction of TG2 expression by TGF-β1-mediated SMAD and TGF-β activated kinase 1 activation of NF-κB ([39] was instrumental in driving the upregulation of Slug, Zeb 1, Snail 1/3, Twist 1/2 with consequent decrease in E-cadherin, which was correlated with mesenchymal phenotype and spheroids formation in OC [27,39]. Similarly, aberrant expression of TG2 in breast cancer (BC) was associated with loss of E-cadherin and upregulation of the transcriptional repressors, Snail1, Zeb1, Zeb2 and Twist1 [37]. In addition, the enzymatically active TG2 protected OC cells from cisplatin-induced apoptosis through activation of the two survival pathways NF-κB and Akt [39]. Of note, treatment of human colon cancer (CC) cell lines with the TG2 selective transamidating inhibitor 1–155 reduced the mesenchymal markers vimentin and FN and the expression of transcription factors Slug and Twist [79]. Consistently, upregulation and activation of TG2 in epidermal squamous cell carcinoma (ESCC) stimulated FAK and Src signaling via α6/β4 integrins [50]. This led to phosphoinositide 3 kinase (PI3K) activation of phosphoinositide dependent kinase 1 (PDK1) which in turn inhibited Hippo signaling, enhanced YAP1 and ΔNp63α accumulation and ECS cell survival. The use of NC9, a TG2’s transamidating inhibitor, disrupted the signaling, thus reducing tumor formation [50].

Remarkably, other studies recognized the TG2’s GTPase activity as a driver of EMT and stemness in BC [38] and ESCC [51]. BC cell lines transfected with GTP-binding-deficient TG2 mutant (R580A) displayed strong overexpression of E-cadherin and a concomitant decrease in mesenchymal markers expression, such as N-cadherin, vimentin, and FN, and the downregulation of transcription repressors Snail1, Zeb 1/2, and Twist 1 compared to cells transduced with wild-type (TG2-WT) or transamidating mutant constructs (TG2-C277S). Similarly, activated NF-κB, Akt and FAK, which support EMT and survival pathways were also decreased in TG2 mutant (R580A) compared to TG2-WT and TG2-C277S [38], strongly suggesting that some of the oncogenic functions exerted by TG2 and previously attributed to the catalytic function, are in fact mediated through the GTP-ase activity. It has also been recognized that some of the TG2 enzymatic inhibitors, such as NC9, alter the conformation of the protein, and indirectly inhibit its GTP-binding pocket [43]. These more recent observations have led to a conceptual shift in attributing the intracellular effects of TG2 away from the transamidating activity to the GTPase function. 

Further understanding of the mechanisms by which TG2 contributes to pro-tumorigenic phenotype will be important to tailor therapeutic targeting.

## 4. TG2/FN/β-Integrin Complex

Resolution of the three-dimensional structure of TG2 indicated that the binding site to FN is located around the β-hairpin loop and mutations within this sequence led to disruption of this complex [8]. It has been recognized more recently that residues outside this also play an important function in mediating the interaction with FN [9]. Reversely, the TG2-binding site on FN has been mapped to a 45-kDa fragment coinciding with the gelatin-binding domain (GBD) (composed of modules I_6_, II_1,2_, and I_7–9_), which mediates the high affinity RGD-independent binding to TG2 [80,81,82]. Further research has restricted this binding site to the C-terminal modules of the GBD [83]. Recently, domain I_8_ was identified as the lesser TG2-interacting module, while domains I_7_ and I_9_ were reported to increase the binding affinity and allow cell function [84]. The complex between TG2 and FN formed on the cell surface stabilizes the direct interactions of both these proteins with integrins, the major adhesion receptors to the ECM [6,85]. The TG2-β1 integrin interaction was detectable in the majority of ovarian tumors and mediated OC stem cells interaction with the tumor niche [12]. Upon targeting the TG2/β1 Integrin/FN complex, the attachment of ovarian cancer cells/spheroids to peritoneal stroma was inhibited [12,52]. In addition, TG2 was found in close association with β1, β4 and β5 integrins on the surface of metastatic MDA-MB231 breast cancer cells where it strongly promoted cell attachment, motility, invasion and resistance to apoptosis [86]. SiRNA-mediated TG2 knockdown significantly blocked MDA-MB231 adhesion to FN-coated surfaces and invasion through matrigel-coated transwell membranes, suggesting that TG2 expression plays an important role in conferring a metastatic phenotype [86]. While little is known about the precise spatial organization of the integrin-TG2 protein–protein complexes, the complementary TG2-FN-binding sites have been delineated, and disruption of this interaction appears to be a promising approach for interfering with cell-ECM adhesion [6]. Efforts to target TG2/FN/integrin complexes have yielded several small molecules with potent activity against cell adhesion to the matrix, initiation of outside-in signaling, and disruption of cancer cell seeding to peritoneal surfaces [52,53]. 

## 5. TG2 in Cancer Stem Cells

The residual small population of CSCs possess has been related to tumor relapse [87]. Their ability to remain quiescent within the tumor microenvironment (TME) and to self-renew allows them to persist during the damaging effects of chemotherapy [88]. The CSC phenotype has been associated with the expression of cell surface receptors, such as CD44 (hyaluronic acid receptor) [89], CD117 (c-Kit) [90], CD133 (prominin-1) [91], or the intracellular activity of the detoxifying enzyme aldehyde dehydrogenase (ALDH) combined along with expression of the cell surface antigen CD133 (ALDH^+^/CD133^+^) [92]. To date, treatment strategies designed to eradicate CSCs remain a significant challenge.

The aberrant expression of TG2 was correlated with CSCs maintenance and spheroids proliferation. In human OC primary cells, TG2 upregulation correlated with the expression of the CSC surface markers CD44^+^ and CD117^+^ [90]. Cao and colleagues demonstrated that TGF-β, a cytokine abundantly increased in the OC microenvironment, regulates NF-κB signaling activation that in turn upregulates TG2 expression, induces EMT, enhancing the percentage of CD44^+^/CD117^+^ cells which is important to cell aggregation as spheroids, the main vehicle of OC tumor dissemination [42]. The data highlighted the interplay between the cellular and non-cellular components in the TME as of critical importance in regulating several aspects of tumor progression, including the maintenance of the CSCs phenotype. 

In agreement with the previous data, Oh and colleagues demonstrated that secretion of the proinflammatory interleukin-6 (IL-6) in the OC milieu was regulated by TG2 primarily through activation of the canonical NF-κB signaling [54]. The TG2/IL-6 axis induction contributed to EMT and the aggregation of OC cells as spheroids, which are critical in the development of peritoneal metastasis. The use of cysteamine as inhibitor of the TG2’s catalytic activity reduced spheroids formation [54]. In human BC, TG2 upregulation was associated with the highly tumorigenic and chemoresistant subpopulation of CSC marked by CD44^+^/CD24^-^ and characterized by self-renewal properties and mammosphere-forming capacity [36]. TG2 expression was also correlated with cell population enriched for glioma stem cells (GSCs) that expresses high levels of CD44 and the inhibitor of DNA-binding 1 protein (ID1) [55]. Furthermore, TG2 knockdown or its pharmacological inhibition by monodansylcadaverine (MDC), a TG2 amine substrate competitive inhibitor, attenuated the expression of ID1 and suppressed tumorigenicity in a glioblastoma (GBM) orthotopic mouse model by blocking the CD44-high GSCs, thus suggesting that inhibition of TG2 transamidating activity might be an effective strategy to block CD44-high GBMs [55]. TG2 was also found to be overexpressed in the phenotypically aggressive and radiation therapy-resistant mesenchymal (MES) subtypes of GSCs compared to the slow-proliferating and less aggressive proneural (PN) phenotype where it regulated the transcription factors (TF) C/EBPβ, PDZ-binding motif-TAZ, and STAT3, critical for maintaining the transcriptome profiling of the MES subtype [56]. The use of GK921, an inhibitor that specifically blocks TG2’s catalytic activity, reduced CD44 and master transcription factor expression levels which in turn blocked cell growth in MES subtype cells and tumor formation in an GBM orthotopic xenograft mouse model [56]. Other data demonstrated a strong link between overexpression of TG2, increased self-renewal and EMT process that regulate the metastatic ability (migration and invasion) of CSCs in CC [57,79]. In addition, the use of 1–155, a TG2 transamidating inhibitor, reduced EMT and spheroid proliferation by blocking the CSC phenotype of CRCs. Conversely, Kerr and colleagues demonstrated the binding of irreversible inhibitors (NC9, VA4, and VA5) at the catalytic site of TG2 promoted a shift from a closed to open conformation affecting the TG2’s GTP-binding site, which in turn decreased ECSC survival and proliferation [43].

ALDH activity regulates the biosynthesis of retinoic acid (RA), reactive oxygen species (ROS) and aldehydes in CSCs [93]. RA is a potent regulator of *TGM2* [94]. Sullivan and colleagues showed that ALDH1A3 and TG2 expression levels were strongly correlated in patient-derived GBM CSCs [44]. Furthermore, ALDH knockdown or inhibition in mesenchymal GSCs led to decreased TG2 expression, and this could be rescued by addition of RA. These results support the existence of a link between retinoid signaling modulated by ALDHs and TG2. 

TG2 was also found to be upregulated in ovarian CSCs identified by the ALDH^+^/CD133^+^ markers combination, compared to non-CSC (ALDH^−^/CD133^−^) [12]. A significant concomitant increase in FN1 and β1 integrin was also observed in CSCs compared to non-CSCs and in proliferative spheroids grown in ultra-low adherence conditions compared to monolayer conditions. A function-inhibiting antibody (clone 4G3) against the TG2 FN-binding domain (1–165) suppressed TG2/FN/integrin complex formation, CSCs proliferation as spheroids, tumor-initiating capacity, and stemness-associated Wnt/β-catenin signaling. This pathway had been already correlated with cancer initiation in other models [95] and with the survival of CSCs in lung adenocarcinoma [96], CR [97], and leukemia [98]. β-catenin was shown to directly regulate expression of the stem cell marker ALDH1A1 in OC cells [99]. Engagement of β-catenin in ovarian CSCs was achieved through the direct interaction between TG2 and the Wnt receptor Frizzled 7. Consistent with these results, Huang and colleagues demonstrated the participation of tissue transglutaminase-1 (TG1), another member of the transglutaminase enzyme family [100], as regulator of stemness and chemoresistance in gastric cancer cells by modulating Wnt/β-catenin signaling [101]. The importance of TG2/FN/β1 Integrin axis was also correlated with progression and metastasis of renal cell carcinoma (RCC) [58]. Downregulation of TG2 led to a decrease in actin stress fiber formation, RCC cells’ adhesion to β1 integrin substrates fibronectin, collagen type I and laminin, and diminished the expression of CD44, CD73-and CD105 CSC markers, supporting that TG2 impacts cancer cell adhesion, migration, invasiveness and cancer cell-stemness during RCC progression and dissemination [58]. 

Altogether, the data indicate that TG2 is highly enriched in CSCs and that its targeting could be developed as therapeutic strategy to eradicate this difficult to treat subpopulation of tumor cells. The multi-functional nature of TG2 with its enzymatic, GTPase, or scaffold properties suggests that the specific inhibition of TG2 in CSCs depends on cell type context and further evaluation of TG2 inhibitors, either alone or in combination with standard chemotherapeutic agents, is warranted in distinct cancer models.

## 6. TG2 in the Extracellular Matrix in Cancer

**Figure 3:**
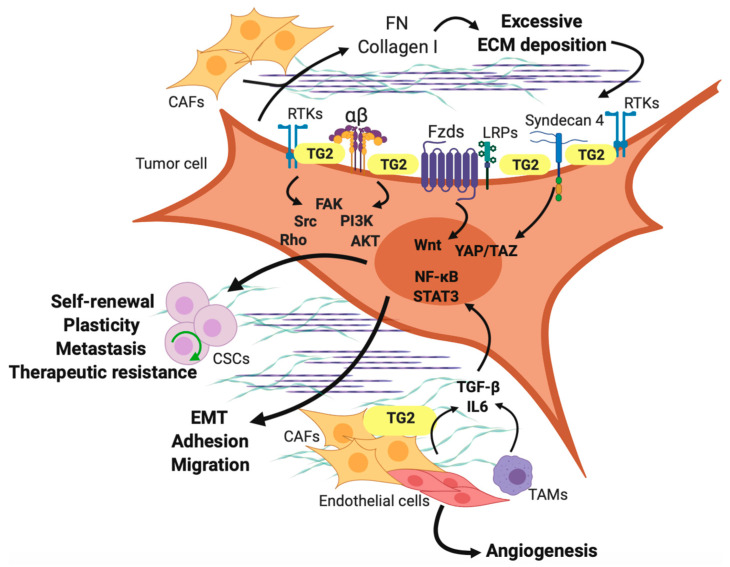
Role of extracellular TG2 in cancer. The different cell populations in the TME, including tumor cells, CAFs, infiltrating immune cells, and endothelial cells, secrete ECM macromolecules (FN and collagen I) and cytokines (TGF-β and IL-6). At the cellular level, TG2 is secreted in the ECM compartment where it interacts with FN, collagen I, TGF-β, and a wide variety of growth factor receptors, integrin β subunits, Wnt receptors, and syndecan-4. These interactions activate intracellular signaling pathways, such as FAK, RhoA, and PI3-K/Akt, and oncogenic signaling (Wnt, YAP/TAZ, NF-κB) involved in cancer initiation, progression, angiogenesis, stemness, and therapeutic resistance.

The roles of TG2 in stromal cells within the TME remain less studied. The TME is composed of several cell types and a complex network of ECM molecules including collagen, FN, laminin, proteoglycans [102]. In this milieu stromal cells secrete growth factors, such as the fibroblast growth factor (FGF), the EGF, the vascular endothelial growth factor/VEGF, as well as cytokines (tumor necrosis factor α/TNF-α, IL6, IL-1β, TGF-β, and others. The growth factors and cytokines provide important cues regulating survival, proliferation, migration and invasion of cancer cells [103]. Given the wide pattern of TG2 expression, including fibroblasts, endothelium, and immune cells, it is important to consider whether its secretion in the TME or its expression in stromal cells alters cancer initiation and/or progression [104]. Within the TME, TG2 has been shown to modulate multiple biological and biomechanical processes, impacting tumor progression and metastasis (Figure 3) [105].

TG2 was shown to be secreted in the ECM not only by cancer cells, but also by fibroblasts, osteoblasts, and endothelial cells through a yet undefined mechanism [106,107]. Once secreted, TG2 is deposited on the plasma membrane and in the ECM where it can function as an enzyme and/or scaffold/adaptor protein, mediating interactions with its binding partners, including integrins [20,85], syndecan-4 [108,109], low density lipoprotein receptor related proteins (LRP) 5 and 6 [109], and several other growth factor receptors and ECM components [107]. In the ECM, TG2 facilitates cell adhesion by connecting the 42-kDa gelatin-binding domain of FN with the β1 and β3 subunits of integrins [6]. As a consequence, cytoplasmic β integrin tails engagement stimulates cell proliferation and metastatic spread regulating intracellular signaling pathways by tyrosine phosphorylation of several protein kinases, such as the FAK and RhoA, involved in the formation of mature focal adhesions complexes and cytoskeleton assembly [31,110]. TG2-mediated resistance to apoptosis induced by chemotherapy has been linked to integrin-mediated signaling converging on PI3-K/Akt pathway, a downstream effector of FAK [39]. Additionally, TG2/FN complex formation on the surface of cancer cell membranes was shown to regulate β-catenin expression and function in OC cells through a c-Src-dependent mechanism [34]. Extracellular TG2 has also been proven to activate the canonical β-catenin signaling by direct binding to the low-density lipoprotein related-protein 5 and 6 (LRP5/6) [109,111]. In an OC model, secreted TG2 was shown to activate non-canonical NF-κB signaling and promote peritoneal metastasis [59]. A specific target of TG2 in this model was the hyaluronan receptor CD44 expressed on cancer cells, which was upregulated in response to TG2 in the peritoneal environment [59]. In A431epithelial carcinoma cells, extracellular TG2 was shown to crosslink the Ca^2+^-binding EF-hand S100 protein S100A4, promoting metastasis [60]. In BC cells, the cell membrane-bound fraction of TG2 was shown to have intrinsic kinase activity and to phosphorylate the insulin-like growth factor-binding protein-3 [112]. Finally, secretion of TG2 in the stroma was reported as being a risk factor for recurrence and poor clinical outcome in breast cancer [61]. Collectively these data support important functions of extracellular TG2 converging at the interface between cancer cells and stroma leading to activation of outside-in signaling.

## 7. TG2 in Cancer-Associated Fibroblasts (CAFs)

Fibroblasts play fundamental roles in wound healing and fibrosis. Fibroblast-derived TG2, acting downstream of TGF-β is important in the effector phase of fibrogenesis in a pulmonary fibrosis mouse model [113]. Upon activation, fibroblasts de-differentiate to a myofibroblastic phenotype characteristic to the wound healing response, as well as to tumor progression when they populate the TME. TG2 was recently proposed as a marker of CAFs in CC, where it is upregulated as compared to normal fibroblasts in patients’ samples, as well as in TGF-β activated fibroblasts [114]. CAFs represent one of the most abundant cell populations in the TME and contribute to tumor growth and dissemination through the release of growth factors and chemokines [115]. CAFs are, however, very heterogenous—diverse fibroblast subpopulations with distinct lineage origin and activation mechanisms have been identified in several solid cancers [116,117,118,119]. Four different CAF subpopulations (named CAF-S1, CAF-S2, CAF-S3, and CAF-S4) were identified by the same group in OC and BC based on multicolor flow cytometry analysis of the TME derived cells using antibodies against key fibroblast markers [120,121]. Specific subsets were shown to play different roles in TME homeostasis—e.g., CAF-S1 is an immunosuppressive fibroblast subpopulation that can be co-targeted for increasing the success of immunotherapy [122]. In addition, CAFs promote alterations of the ECM components that support tumor behavior. In fact, many solid tumors show a different profile of matrix proteins, including collagen, laminin, hyaluronan, and FN than their normal counterparts, and many of these proteins interact directly with tumor cells through integrins or cell surface receptors, regulating cancer cell functions, such as proliferation, apoptosis and migration [123]. The expression of TG2 in fibroblasts (Figure 3) was correlated with ECM re-organization, cell adhesion, and migration in both physiological and pathological conditions. Expression of TG2 in CAFs and in the desmoplastic stroma associated with pancreatic cancer was linked to poor clinical prognosis [48]. TG2 mediated crosstalk between cancer cells and fibroblasts was reported in pancreatic tumors, where (enzymatically active) TG2-expressing cancer cells corrupted fibroblasts to increase collagen matrix deposition, which further promoted cancer cell proliferation by stimulating YAP/TAZ signaling [48]. In hepatocellular carcinoma, CAFs were shown to induce EMT in cancer cells in a TG2-dependent manner, mediated by IL-6/IL6R/STAT3 axis [62].

## 8. TG2 in Immune Cells

Indirect evidence of TG2 involvement in inflammation was provided by several studies using inflammatory disease models. Two reports have shown decreased inflammation in animal models of allergic conjunctivitis [124] and lipopolysaccharide (LPS)-induced lung inflammation [125], upon treatment with octapeptide R2 (KVLDGQDP) targeting TGase activity. In allergic asthma models, TG2 induced the expression of numerous signaling molecules associated with airway inflammation and remodeling [126]. One of the mechanisms by which TG2 modulates inflammatory responses is by activating phospholipase A2 (PLA2)—a family of lipolytic enzymes responsible releasing inflammation mediators from membrane lipid storage sites [124,126,127,128]. In an in vitro model of LPS-mediated macrophage activation, TG2 was found to interact with the secreted isoform of PLA2 group V (sPLA2-V) [128]. A fourfold increase in *TGM2* gene expression and twofold TG2 protein increase was observed at 24 h post LPS treatment, consistent with a 1.5-fold increase in sPLA2 protein expression. Confocal microscopy and co-immunoprecipitation analyses revealed a TG2-sPLA2 complex in THP-1 cells, suggesting that sPLA2 may act as a substrate for amine incorporation mediated by TG2. This conclusion is supported by previous studies where PLA2 polyamination was linked to inflammation [129]. Additionally, previous reports have demonstrated that TG2-catalyzed post-translational modification of porcine pancreatic PLA2 leading to its increased activity via either intramolecular crosslinking at Gln-4 followed by noncovalent dimerization [130] or incorporation of polyamines [131]. Sustained activation of PLA2 leads to eicosanoid production of pro-inflammatory lipid mediators. Interestingly, TG2-mediated mast cells and macrophages interaction was shown to enhance metastatic potential of mouse melanoma tumor cells [63].

Within the TME, the interaction between the immune system and tumor cells is much more complex than initially assumed by the theory of immune surveillance and protection against tumor development depends not only on the adaptive, but also on the innate immune response [132]. Tumors can block the development of an adequate immune response both by acting directly on the cells of the immune system (direct action) or by recruiting cells capable of exercising immunosuppressive activity (indirect action) [132,133]. Cancer cells also secrete various soluble factors, which recruit and expand populations of regulatory T lymphocytes, immature dendritic cells, tumor-associated macrophages (TAMs) and myeloid-derived suppressor cells, which inhibit both innate and adaptive immune responses [134]. Several reports indicate that TG2 is involved in cell-mediated immunity and its expression has been found in macrophages, dendritic cells (DC), myeloid cells, T and B cells. Interestingly, high TG2 levels are present in a common clonogenic bone marrow progenitor specific for macrophages and DCs [135,136]. TG2 is upregulated during the functional maturation of DCs from monocytes after LPS treatment [137] and it secretion on the surface of DCs mediates the interaction between DC and T cells promoting the adaptive immune response [138]. KCC009, an enzymatic TG2 inhibitor was shown to impair the function of DCs leading to reduced secretion of cytokines (IL-10, IL-12) and consequently decreased production of IFN-γ by T cells [137]. Experiments carried out in TG2^−/−^ mice showed impaired DC maturation [137] and decreased numbers of memory T cells [64]. TG2 was shown to regulate phagocytosis of apoptotic debris and necrotic cells by macrophages by mediating the formation of the phagocytic cup [139], thereby perpetuating a pro-inflammatory milieu. In macrophages, TG2 also binds MFG-E8 (milk fat globulin EGF factor 8), a protein known to bridge β3 integrin to apoptotic cells during phagocytosis [139]. Macrophages from TG2^−/−^ mice showed impaired phagocytosis [139,140] and responded to LPS with a proinflammatory phenotype characterized by elevated IL-6 and tumor necrosis factor-α (TNF-α) secretion [141]. Interestingly, TG2 targeting with siRNA-mediated knock-down or with R283, a specific TG2 enzymatic inhibitor, reduced the inflammatory processes by downregulating markers of monocyte maturation (CD14, FN), and activation of activator protein-1 (AP1)/c-Jun N-terminal kinase (JNK) signaling, TNF-α and matrix metalloprotease-9 (MMP-9) [142,143].

The mechanism by which TG2 expression in immune cells affects cancer progression has not been extensively characterized. In pancreatic ductal adenocarcinoma (PDAC), TG2 expression was detected among tumors profiled as representing an “immunosuppressive” phenotype associated with poor clinical outcome. This subgroup contained higher number of M2 macrophages and T regulatory cells and decreased numbers of pro-B and memory B cells [144]. TG2 upregulation was strongly correlated with the expression of the exhaustion marker PD-L1. While a mechanism was not clearly defined, it was proposed that TG2 may be regulating PD-L1 through activation of STAT3 and NF-κB signaling [144]. In gastric cancer, TG2 upregulation was shown to enhance inflammation and promote tumor growth by recruiting macrophages to the tumor milieu via Il-1β-mediated induction of CCL2 and CXCL10 [65]. Given that TG2 is also a marker of tumor promoting M2 polarized TAMs [145], therapeutic targeting of TG2 could both target cancer cells, as well as pro-inflammatory processes supported by macrophages. Recent work in PD-L1 inhibitor resistant triple negative BC patients revealed that inhibition of TG2, restored T cell-dependent cytotoxicity by inhibiting expression of PD-L1 and CCL2 in PD-L1^+^ breast cancer cells [66].

In OC, the function of TG2 expressed by the host tissue was investigated by using the Roby syngeneic OC model [146]. Tumor dissemination was delayed and ascites accumulation was inhibited in TG2^−/−^ mice [67]. The peritoneal TME was infiltrated by activated cytotoxic T cells and T cell differentiation to an effector/memory fate was promoted in TG2^−/−^ mice. Moreover, TG2^−/−^ mice showed decreased immunosuppressive TMEs: less TAMs infiltration and decreased PD-L1 expression in myeloid and cancer cells in the ascites of tumor bearing hosts [67]. TAMs are known to support ascites spheroids formation [68] and TG2 is a marker of M2 pro-tumorigenic TAMs [145]. Image cytometry quantification of ovarian tumor samples revealed that high TG2 expression in the stroma was inversely correlated with CD8^+^ T cells infiltration [67]. Given the overall survival in OC is strongly correlated with the numbers of tumor infiltrating lymphocytes (TILs) [147], the data suggest that one mechanism by which TG2 influences cancer progression may be linked to modulation of numbers and function of cytotoxic CD8^+^ T cells. Collectively, these recent reports support that TG2 expression in immune cells could play an important function fine tuning tumor progression; however, additional studies are needed to fully elucidate the involved pathways.

## 9. TG2 in Endothelial Cells

Angiogenesis is a hallmark of cancer and required to sustain tumor growth and dissemination. The angiogenic process is initiated through the activation of endothelial cells, stimulated by cytokines released from tumor cells and the other cellular components of the TME as a result of the hypoxic microenvironment [148]. Hypoxic tumor cells, macrophages, and other cells of the immune system, stimulate the production of hypoxia-inducible factor 1 α (HIF-1 α) which induces upregulation of angiogenic factors such as VEGF, FGF, PDGF, and TGF-β, causing blood vessel growth, increase in vascular permeability and release of proteases, important for cancer cell invasion and metastatic dissemination [149]. It has been established that when secreted in the TME, TGF-β induces the expression and function of TG2 which in turn activates HIF-1α and NF-κB signaling via the non-canonical pathway [42,49]. This evidence in correlation with a TGF-β/HIF-1α/NF-κB-mediated VEFG activation suggests a potential involvement of TG2 in angiogenesis. In fact, TG2 has been found highly expressed in endothelial cells and its crosslinking activity in the extracellular compartment promoted VEGF receptor 2 (VEGFR2)-dependent sprouting angiogenesis [69,150,151]. In addition, TG2 was shown to regulate the activation of matrix-bound TGF-β1 signaling, which is also required for VEGFA-mediated endothelial tubule formation [70].

In the endothelium, the extracellular pool of TG2 is involved in regulating cell–matrix adhesion, while the cytoplasmic TG2 is important for cell cycle progression and cell survival [71]. Loss of TG2 in endothelial cells could affect cell numbers by inducing cell cycle arrest and/or promote apoptosis caused by defective cell adhesion to the matrix. Contradictory results, however, have been reported regarding the mechanism of action of TG2 and its inhibitory or stimulatory effect in angiogenesis (Figure 3). TG2 in complex with integrins regulates signal transduction which activates fibroblasts and endothelial cells, thereby promoting angiogenesis [69]. Conversely, TG2 crosslinks matrix fibers which was suggested to inhibit it [23]. However, TG2 knockout animals manifested no significant vascular phenotype compared to wild-type mice [152], possibly due to compensatory mechanisms involving other TGs [153]. Using in vitro and in vivo angiogenesis models, Wang Z et al. have shown that extracellular TG2 transamidation activity, but not the GTP-binding activity, is essential during tubule formation and branching [69]. TG2 inhibition greatly reduced matrix-bound VEGF and ECM FN and pro-angiogenic signaling via VEGFR2. Additionally, TG2 was found to be important in human umbilical vein endothelial cells (HUVEC) migration by mediating the interaction of VEGFR2 with β1 integrin. Interestingly, the active enzyme becomes inhibitory at higher concentrations (1 μg/mL), which reconciles these findings with apparently contradictory reports [23] highlighting TG2 as angiogenesis and tumor growth inhibitor. Further work from the same group showed that exogenous addition of TGF-β 1 (in pg/mL range) partially rescues the loss of tubule formation phenotype observed upon TG2 inhibition [70]. Increased extracellular TG2 or TGF-β 1 induced p-Smad2/3 signaling and endothelial-mesenchymal transition, demonstrated by the loss of CD31 and VE-cadherin expression and increased levels of mesenchymal markers, such as vimentin, α-SMA, S100A4, and FN. This leads to dysfunction of blood vessels, known as capillary rarefaction, encountered in pathological states such as fibrosis [154]. Similar to VEGF, inhibition of TG2 crosslinking activity reduced matrix-bound TGF-β 1. Previous reports showed a role of TG2 in the activation of matrix-bound TGF-β 1 via crosslinking of the latent TGF-beta binding protein (LTBP) [155].

TG2 was detected at both the cellular and tissue levels in gastric cancer and high TG2 expression correlated with poor prognosis in gastric cancer patients [72]. The peptide GX1 inhibited TG2’s GTP-binding activity thereby suppressing angiogenesis through downregulation of HIF-1α/NF-κB axis in gastric cancer endothelial cells [72]. In renal cell carcinoma, TG2 enzymatic activity promoted proangiogenic response by activating HIF-1α through the degradation of p53. Mechanistically, TG2-mediated suppression of p53 allowed the HIF-1α–p300 interaction with subsequent VEGF upregulation [72]. In bladder carcinoma, TG2 was found to be involved in vascular mimicry of carcinoma cells, which can be independent of VEGF [156]. Thus, a variety of processes are affected by TG2 in endothelial and stromal cells altering angiogenesis and impact tumor growth.

## 10. TG2 and Matrix Metalloproteases in Metastatic Progression

To initiate the formation of new capillaries, the EC of existing blood vessels must degrade the basement membrane (BM) and invade the stroma of the adjacent tissue. This process requires cooperation between the plasminogen activator system (PA) and that of the MMPs, in particular MMP-1, MMP-2, MMP-3 e MMP-9 [157,158]. MMPs are a family of proteolytic enzymes that digest all components of the ECM [159]. Under normal physiological conditions, the process of connective tissue remodeling by MMPs is strictly regulated. In the context of malignancy, uncontrolled remodeling leads to degradation of the ECM and breakdown of the basement membrane at the leading edge of a tumor, thus facilitating cancer cell invasion and metastatic spread [160,161].

In human cancer, FN has been identified as major glycoprotein secreted by tumor cells and its aberrant expression has been correlated with MMPs secretion and poor prognosis [162,163]. Mechanistically, the interaction between TG2, FN and the β integrins is followed by MMPs secretion and activation. Aberrant expression of membrane type (MT)-MMP led to proteolytic degradation of cell surface TG2 at the leading edge of motile glioma and fibrosarcoma cells, thereby suppressing cell adhesion and migration on FN [73]. Of relevance, proteolysis of TG2 colocalized with MT-MMPs was prevented in cells cultured on FN matrix, supporting cell adhesion and locomotion. Furthermore, MMP-2-mediated cell surface TG2 degradation in fibrosarcoma cells inhibited its enzymatic function [164]. In OC, decreased TG2 expression was correlated with decreased expression of MMP-2, FN, and other critical mediators of metastasis [74]. Mechanistic in vitro and in vivo studies demonstrated that TG2-dependent degradation of protein phosphatase 2 (PP2A-α) activated the expression of MMP-2 in a cAMP-response element-binding protein (CREB)-dependent fashion, leading to cancer progression [74]. In squamous carcinoma, the highly invasive A431-III cancer cell subline was compared to the parental cells (A431-P) and were shown to have increased adhesion, spreading, migratory, and invasive properties [75]. Knockdown of TG2 by siRNA dramatically reduced cell attachment, migration and invasion, and the secretion of MMP-9 and MMP-1 (but not of MMP-2 and MMP-3) in A431-III cells as compared to A431-P cells. Furthermore, knockdown of TG2 markedly suppressed β1 integrin interaction with FN [75]. These data support the effects of TG2 on cancer cell invasiveness through modulation of MMP functions.

## 11. TG2 and the Stiff Matrix

Tumor cell behavior, including signaling, proliferation, migration and invasion depend on the mechanical forces and biochemical signals generated from the interaction of single cancer cells and the surrounding ECM environment [165]. Increased ECM stiffness is observed in both metastatic carcinoma cell lines and primary tumor cells compared to non-cancerous counterparts in in vitro and in vivo models of cancer [166]. Mechanistically, ECM stiffness is a feature of most solid tumors, regulates the expression of pro-metastatic genes, and correlates with progression from normal epithelium to malignancy [167]. One of the major contributors to the stiff stroma is the increased deposition of fibrillar collagen I that strengthens the ECM, organizes it, makes it flexible and ensures its resistance to traction [168]. TG2 mediates crosslinking of collagen I increasing its deposition in fibrotic disorders and in cancer [169].

In PDAC, the aberrant proliferation of stromal cells, such as myofibroblasts and pancreatic stellate cells, and secreted collagens promote cancer growth, metastasis, and drug resistance [170]. TG2 was shown to be abundantly expressed and active in pancreatic tumors [48]. When secreted in the pancreatic TME, TG2-mediated collagen crosslinks, stimulated the proliferation of myofibroblasts, which in turn activated the YAP and TAZ transcription factors in cancer cells, promoting PDAC cell proliferation. TG2 knockdown inhibited the growth of orthotopic pancreatic xenograft models [48]. In addition, TG2 was shown to alter the response to gemcitabine by facilitating the interaction between PDAC and stromal cell, [35]. This occurred as a consequence of decreased laminin secretion from fibroblasts in PDAC xenografts derived from TG2 knock down cells, contributing to increased sensitivity to gemcitabine. Similar results were observed in pancreatic-tumor-bearing mice treated with gemcitabine with or without TG2-specific siRNA-DOPC (1,2-dioleoyl-sn-glycero-3-phosphatidylcholine) liposomes [46]. In BC, human mammary epithelial cells (HMLE) overexpressing TG2 showed tissue stiffening via robust FN and collagen accumulation that was associated with advanced disease progression at both the primary tumor and metastatic sites [171]. Collagen deposition and remodeling shape the desmoplastic stroma in CC with a profound impact on disease progression [172]. In this context, Delaine-Smith and colleagues demonstrated in a collagen gel 3-D co-culture system of fibroblast and CRC cells that TG2 induced formation of thicker collagen fibers, which was correlated with tissue stiffening and associated with a poor outcome in CRC patients [76].

Furthermore, recent studies suggest that a stiff ECM is associated with biomechanical properties, such as stress and force distribution profiles, and reorganization of the cytoskeleton that favor collective migration of epithelial-like tumor cells [173]. In this regard, EGF signaling through Ras and c-Jun N-terminal kinase (JNK) increased the transamidating and GTPase functions of TG2, as well as its accumulation along the leading edges of actively migrating cervical carcinoma cells [77]. TG2 targeting by either siRNA-mediated knockdown or by using MDC reduced the stimulating effects of EGF on cell migration and invasion [77]. In all, these reports show the potential importance of TG2 as a key regulator of the changes in the ECM and the activation of mechano-sensor pathways in response to the increased stiffness observed in cancer. This is of particular importance as it defines TG2 as a potential target for therapeutics targeting tumor–stroma interactions.

## 12. Conclusions

A ubiquitous protein with complex functions regulated by distinct tissue contexts, TG2 stands at the interface between tumor cells and stroma guiding cancer cell behavior under difficult circumstances. By and large recognized as a pro-inflammatory and pro-tumorigenic protein, TG2 has remained an elusive target. Although several strategies have been pursued, including blockade of the TG2/FN complex formation and inhibition of the GTPase activity or of the enzymatic core, none have yielded convincing results. As the field evolves, efforts towards developing a strategy able to inhibit all of the protein’s functions should become a priority.

## Figures and Tables

**Table 1:** References describing TG2 involvement in cancer processes.

References	Type of Cancer Model	Oncogenic Signaling	Adhesion and Migration	ECM Remodeling and Invasion	EMT	Metastasis	Angiogenesis	Stemness	Chemotherapy/Radiotherapy Resistance	(Anti-Tumor) Immune Response
Condello (2018), [12]	ovarian cancer stem cells and tumors							●		
Jones (2005), [23]	CT26 colon carcinoma tumors			●			×			
Kleman (1995), [24]	rhabdomyosarcoma cells			●						
Satpathy (2007), [26]	peritoneal ovarian tumors					●				
Shao (2009), [27]	ovarian tumors				●					
Verma (2006), [31]	pancreatic ductal adenocarcinoma (PDA)					●			●	
Hwang (2008), [32]	ovarian carcinoma cell lines; in vivo chemotherapy-sensitive (HeyA8) and chemotherapy-resistant (HeyA8-MDR and RMG2) models			●			●		●	
Jeong (2013), [33]	non-small cell lung cancer patients								●	
Condello (2013), [34]	ovarian cancer cells and tumors	●								
Lee (2015, 2016), [35,48]	orthotopic pancreatic xenografts and co-culture of PDA and stromal cells;	●		●					● (TMA secreted TG2 crosstalk with pancreatic cancer-associated fibroblasts; × (PDA cells)	
Kumar (2010, 2011, 2012), [36,37,38,49]	human mammary epithelial (MCF10A), breast cancer MCF7, and drug-resistant MCF7-RT cells	●		●	●			●	●	
Cao (2008), [39]	Epithelial ovarian cancer cells	●							●	
Mann (2006), [40]	pancreatic ductal carcinoma	●								
Mehta (2004), [41]	metastatic breast cancer cell line MDA-MB-231 and subclones; primary vs. metastatic lymph node breast cancer tumors			●		●			●	
Cao (2012), [42]	ovarian cancer cells	●			●	●		●		
Kerr (2017), [43]	squamous cell carcinoma—SCC-13 cells	●						●		
Fisher (2016), [50]	squamous cell carcinoma—SCC-13 cells	●						●		
Fisher (2015), [51]	squamous cell carcinoma—SCC-13 and A431 cells				●			●		
Sullivan (2017), [44]	proneural vs. mesenchymal glioma stem cells	●						●	●	
Verma (2008), [45,46]	pancreatic cancer cells; athymic nude mouse model; orthotopic PDAC tumors in nude mice; stage II PDAC patient samples	●	●			●	●		●	
Singh (2001), [47]	HeLa endometrial cancer cells	●	●							
Sima (2019), [52]	ovarian cancer cells; in vivo model measuring intraperitoneal dissemination		●						●	
Yakubov (2014), [53]	SKOV3 and IGROV1 ovarian cancer cells		●	●						
Oh (2015), [54]	human ovarian cancer cells	●				●		●		
Fu (2013), [55]	glioma-initiating cell lines from fresh surgical glioblastoma samples							●		
Yin (2017), [56]	xenograft mouse model of glioma	●			●				●	
Kang (2018), [57]	human colorectal cancer cells—TU12 cell line derived CSCs subpopulations				●	●		●		
Bagatur (2018), [58]	Caki-2 and A-498 primary site and Caki-1 and ACHN metastatic site renal cell carcinoma cell lines		●			●		●		
Yakubov (2013), [59]	i.p. and orthotopic ovarian cancer xenografts	●		●	●	●				
Biri (2016), [60]	A431 epithelial carcinoma cells		●			●				
Assi (2013), [61]	stroma of breast invasive ductal carcinomas vs. normal breast tissue			●						
Jia (2020), [62]	hepatocellular carcinoma cells	●			●					
Eom (2014), [63]	B16F1 mouse melanoma cells, in vitro and in vivo	●				●	●			●
Kim (2014), [64]	in vivo mouse T cells—contact hypersensitivity reaction; ex vivo restimulation of spleen T cells with tumour lysate-loaded wild-type dendritic cells from immunized mice									× (increased effector and CD8^+^ memory response)
Cho (2020), [65]	gastric cancer									● (tumor-promoting inflammation)
Choi (2020), [66]	triple negative breast cancer	●								● (PD-1/PD-L1 inhibitor-resistance)
Sima (2021), [67]	ovarian cancer syngeneic TG2 null mouse model	●								● (decreased CD8^+^ mediated anti-tumor immune response)
Yin (2016), [68]	tumor-associated macrophages from ovarian cancer									● (promotion of intraperitoneal spheroid formation)
Wang (2013), [69]	HUVEC cell culture, aorta ring assay and in vivo angiogenesis models	●					●			
Wang (2017), [70]	endothelial cells (ECs) and fibroblast co-culture and ECs 3D culture models	●			●		●			
Nadalutti (2011), [71]	endothelial cells		●							
Lei (2018), [72]	Tumor endothelilal cells from gastric cancer						●			
Belkin (2001), [73]	glioma and fibrosarcoma cells		●	●						
Satpathy (2009), [74]	ovarian cancer cells			●						
Chen (2010), [75]	A431 epithelial carcinoma cells			●						
Delaine-Smith (2019), [76]	organotypic 3D fibroblast/SW480 co-culture models of colorectal cancer			●						
Antonyak (2009), [77]	HeLa carcinoma cells, highly aggressive breast cancer cell line MDAMB231	●	●	●						

Symbols ● or × mark if data support (●) or not (×) TG2 pro-tumorigenic involvement in those specific processes.

## Data Availability

Not applicable.
